# Functional Outcomes and Quality of Life after Restorative Proctocolectomy in Paediatric Patients: A Case-Control Study

**DOI:** 10.1155/2014/340341

**Published:** 2014-03-16

**Authors:** Gianluca Pellino, Guido Sciaudone, Erasmo Miele, Giuseppe Candilio, Gilda Serena De Fatico, Gabriele Riegler, Annamaria Staiano, Silvestro Canonico, Francesco Selvaggi

**Affiliations:** ^1^Unit of General Surgery, Second University of Naples, Piazza Miraglia 2, 80138 Naples, Italy; ^2^Department of Paediatrics, University of Naples Federico II, Italy; ^3^Unit of Gastroenterology, Second University of Naples, Italy

## Abstract

*Background.* Restorative proctocolectomy with ileal-pouch anal anastomosis (IPAA) has some peculiarities in paediatric ulcerative colitis (UC). *Aims*. The primary aim was to compare the bowel function of patients undergoing IPAA between those operated on in childhood and adulthood. The secondary aim was to compare the quality of life (QoL) and outcomes for children between medical and surgical therapies. *Method*. Children undergoing IPAA were compared with adult patients undergoing IPAA between 2007 and 2012. Function was assessed 1 year after ileostomy closure. Function and QoL of medically managed paediatric patients were compared with their surgical counterparts. *Results*. Twelve paediatric IPAA patients were compared with 24 adult ones. Acute presentation was common in the former, usually after failed biological treatment. Recurrent pouchitis was more frequent in children. Younger patients exhibited a trend toward better discrimination and continence. QoL was excellent in both groups. Twelve medically treated children were enrolled for secondary aim. Functioning was similar in IPAA- and medically managed children, but the former had a better QoL, confirmed by parents' perception. *Conclusions*. Similar function is achieved by IPAA in childhood or adulthood. IPAA may offer a better QoL compared to prolonged medical management. The beneficial effects of IPAA experienced by children were similarly observed by their parents.

## 1. Introduction

Restorative proctocolectomy involving the formation of an ileal pouch and ileal-pouch anal anastomosis (IPAA) is the treatment of choice for refractory or complicated ulcerative colitis (UC) [1]. IPAA is regarded as the mainstay treatment for UC in both the elderly [2] and the paediatric population [3]. However, this is a very complex procedure, and inadequate data exist comparing the functional outcomes of patients undergoing IPAA for UC between those operated on in childhood and adulthood. The available reports are difficult to interpret because of disparities in patient assessments and the wide range of observations [4]. The primary aim of this study was to compare the functional outcomes of IPAA between a paediatric cohort and adult patients. The secondary aim was to compare the bowel function and quality of life (QoL) of children diagnosed with UC between those undergoing IPAA and those treated medically.

## 2. Materials and Methods

The data of patients diagnosed with UC and treated or referred to our institutions between 2007 and 2012 were collected prospectively.

### 2.1. Primary Aim

The data of 12 patients aged between 5 and 16 years undergoing IPAA for UC during the study period were collected and compared with those of patients aged between 30 and 45 years of age (ratio 1 : 2). The patients in each group were matched according to sex, disease duration, and surgical details.

The surgical treatment was performed as a two- or three-stage procedure in an elective or emergency setting, respectively, and, in the case of paediatric patients, always required fashioning of a stoma at the time of IPAA [5–7]. Adult patients undergoing one- or two-stage modified IPAA surgery were not included. The operative variables and complications were evaluated in the perioperative phase, within 30 days of the IPAA. Functional outcomes were assessed at the 1-year follow-up after ileostomy closure, which was carried out after clinical and endoscopic evaluations. A pouchography was performed in selected cases [7]. Patients were asked to complete a 7-day diary of bowel movements. QoL was assessed 1 year after ileostomy takedown using the age-adjusted Pediatric Quality of Life Inventory (PedsQL) Short Form-15 (SF-15) [8] and the inflammatory bowel disease questionnaire (IBDQ) [9] for the children and adults, respectively.

### 2.2. Secondary Aim

The data of patients aged between 5 and 16 years undergoing IPAA for UC (*n* = 12) were compared with those of consecutive age-matched patients with UC who were managed medically (*n* = 12). Patients serving as controls were included, if they had been diagnosed with UC at least 1 year previously.

Bowel control and symptoms were graded according to the PedsQL Gastrointestinal Symptom Scale (PedsQL-GSS) [10], which was administered to the patients and parents of both groups. QoL was evaluated using the PedsQL-SF-15 in both groups 1 year after ileostomy closure and after the first medical treatment.

### 2.3. Statistical Analysis

The results are expressed as median (range) or *n* (%) values, as appropriate. Differences in medians between subgroups were compared using the Mann-Whitney *U* test. Comparisons between categorical variables were analysed using Fisher's exact test. The cutoff for statistical significance was set at *P* < 0.05. Data were analysed using the SPSS statistical package (version 17.0, SPSS for MS Windows, Chicago, IL, USA).

## 3. Ethical Considerations

This study was conducted following the Guidelines for Good Clinical Practice and was approved by the internal Institutional Review Board. Written informed consent to participate was provided by all of the paediatric patients' parents and by all of the adult patients serving as controls.

## 4. Results

Twelve patients who underwent IPAA for UC during the study period were included in the study. Twenty-four adult UC patients with IPAA and 12 age-matched, paediatric UC patients who were managed with medical treatment during the study period served as controls for the primary and secondary aims, respectively.

### 4.1. Primary Aim

The demographic, clinical, and surgical data of the IPAA patients are reported in Table 1, sorted according to the age at surgery. Although the adult patients appeared more likely to be receiving immunosuppressant drugs at the time of first surgery and on treatment with drugs for conditions other than UC when compared with the younger patients, the difference was not statistically significant. Conversely, a three-stage approach was carried out significantly less frequently among the adult patients.

Patients undergoing IPAA in childhood were more often found with extra-intestinal manifestations (EIMs; *P* = 0.02). More than half of the adult patients but less than one-quarter of the children underwent surgery without receiving treatment with biological drugs (*P* = 0.4).

No significant differences were observed in terms of major perioperative complications (one child suffered a haemorrhage and one adult had an anastomotic leak; *P* > 0.99).

Three patients in each group experienced at least one episode of pouchitis at the 12-month follow-up (25% versus 12.5%, *P* = 0.38); all three of the paediatric patients with pouchitis experienced recurrent pouchitis, compared with one of the adult cases (*P* = 0.09). All cases of pouchitis were managed with antibiotic administration.

**Figure 1:**
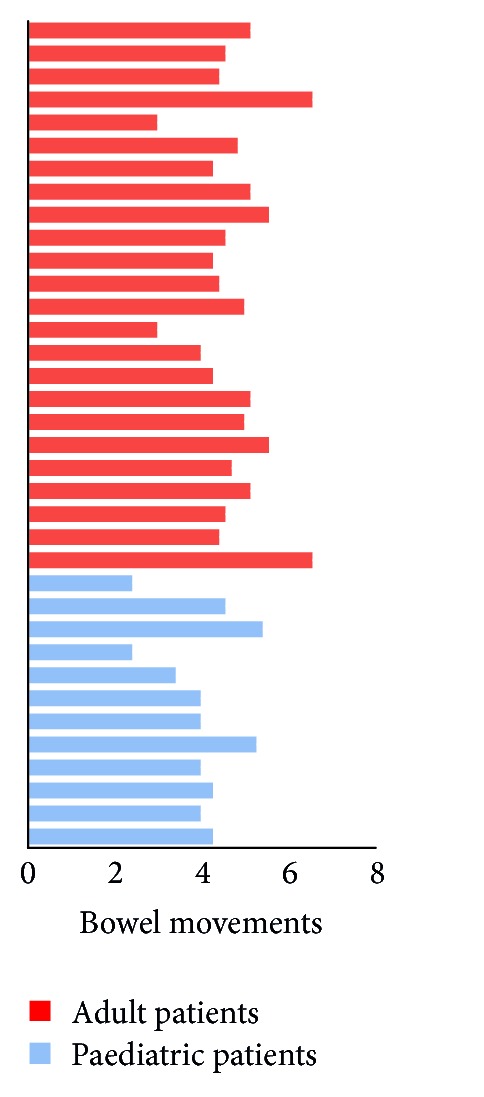
Stool frequency in adult and paediatric patients with IPAA. Bars represent the mean scores of a 7-day diary. Most patients reported four to six bowel movements daily. No significant differences were observed relative to age at surgery [paediatric versus adult IPAA, median (range): 5 (1–9) versus 5 (2–9), *P* = 0.54].

The functional results are given in Table 2. The median stool frequency was similar in the two groups, although the younger patients reported fewer bowel movements per day in their 7-day diaries (Figure 1). No significant differences were observed in patients with night-time incontinence and urgency, and although the younger patients exhibited a trend toward better discrimination (good discrimination in 92% of younger patients versus 80% of controls) and daytime continence (100% versus 96%), the difference did not reach statistical significance. Irrespective of the age at surgery, less than 35% of the patients in each group had a suboptimal QoL.

### 4.2. Secondary Aim

Table 3 lists the characteristics of the patients according to the treatment delivered. Twelve medically treated UC children served as controls. Although the IPAA patients appeared to have a lower body mass index (BMI) and higher rates of EIMs and treatment with biological drugs, the groups were actually statistically homogeneous.

**Figure 2:**
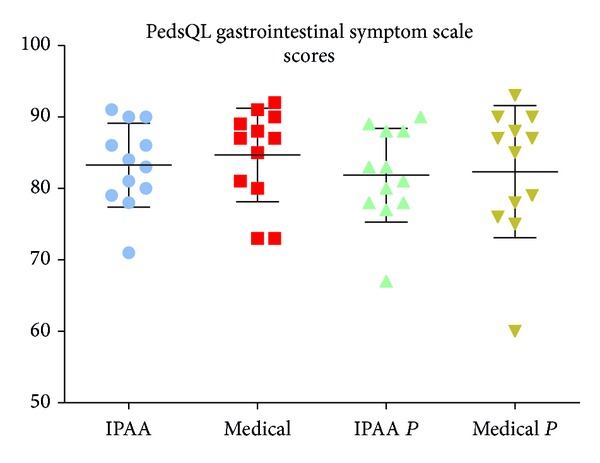
PedsQL-Gastrointestinal Symptom Scale scores in children receiving either surgical (IPAA, *n* = 12) or medical (medical, *n* = 12) therapy. The parents' scores for the two groups are reported as IPAAp and medicalp, respectively. No differences were observed: IPAA versus medical, 0.83 versus 0.87 (*P* = 0.42); IPAAp versus medicalp, 0.82 versus 0.86 (*P* = 0.79); IPAA versus IPAAp, 0.83 versus 0.82 (*P* = 0.5); medical versus medicalp, 0.85 versus 0.82 (*P* = 0.62). IPAA: ileal pouch-anal anastomosis; p: parent.

The PedsQL-GSS revealed no differences in function or bowel control (Figure 2). The IPAA and medical patients achieved scores of 0.83 (0.71–0.91) and 0.87 (0.73–0.92), respectively (*P* = 0.42). Furthermore, the parent-reported that scores did not differ significantly between the two groups [0.82 (0.90–0.67) versus 0.86 (0.93–0.60), *P* = 0.79]. There was good agreement between the patients' and their parents' reports.

**Figure 3:**
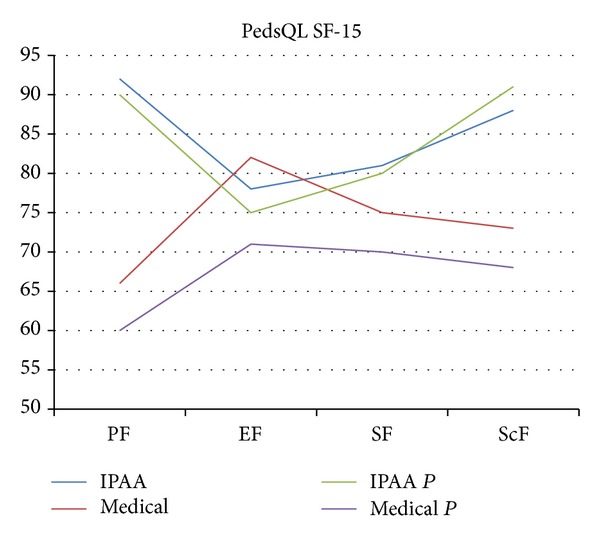
PedsQL-SF-15 scores of IPAA and medical patients. Each dimension of the questionnaire is reported separately. Several significant differences were observed. Overall QoL-IPAA versus medical, 0.84 versus 0.74 (*P* = 0.04); SF-IPAA versus medical, 0.92 versus 0.68 (*P* = 0.02); ScF-IPAA versus medical, 0.88 versus 0.72 (*P* = 0.04); overall QoL-medical versus medicalp, 0.72 versus 0.67 (*P* = 0.04). PF: physical function; EF: emotional function; SF: social function; ScF: school function; IPAA: ileal pouch-anal anastomosis.

The overall median QoL was higher for the IPAA patients than for the medically treated patients (0.84 versus 0.74, *P* = 0.04; Figure 3). Comparison of each dimension of the PedsQL-SF-15 revealed that the IPAA patients scored significantly higher than their medically managed counterparts in physical functioning (0.92 versus 0.68, *P* = 0.02) and school functioning (0.88 versus 0.72, *P* = 0.04). The parents of both groups reported lower scores compared with the patients; a statistically significant discrepancy was observed in medically treated patients, with their parents reporting worse QoL scores overall (0.72 versus 0.67, *P* = 0.04).

## 5. Discussion

The present data suggest that there are some age-related differences in the presentation and preoperative management of patients suffering from UC. IPAA can be safely performed in childhood by experienced surgeons to produce functional results that are comparable to those of patients undergoing the pelvic pouch procedure in adulthood. Irrespective of age, an excellent QoL can be expected in more than half of the patients undergoing IPAA; the results on continence and discrimination of the procedures performed at an earlier age are promising. However, refractory/recurrent pouchitis may be an issue.

Children diagnosed with UC who underwent IPAA had more similar bowel control than age-matched, medically managed controls in this study. IPAA patients may achieve higher QoL scores by 1 year after ileostomy closure and once they have adapted to the new function, a finding that was reflected by the parents' perception of their children's QoL.

The aims of the treatment of paediatric patients affected with active UC are to induce and maintain remission, improve QoL, ensure normal growth, and prevent colonic neoplastic degeneration [5]. Failure to gain weight, the significant side effects of corticosteroids and long-standing disease are peculiar indications to paediatric IPAA surgery [11]. Children more frequently present with pancolitis and a more aggressive disease course [3]. In the present series, the incidence of EIMs at disease onset was higher among the young patients than among their adult counterparts. Children more often required subtotal colectomy because of aggressive disease (Table 1) associated with intense activity of the immune system, with early medical refractoriness or rapid worsening of health status [4]. Biological drugs are useful for postponing surgery, thus allowing elective colectomy, and may be used to avoid immunosuppressive therapy [3, 12]. When compared with the adult IPAA patients, those undergoing IPAA in childhood were more likely to be receiving a rescue therapy with biological drugs, failing to avoid surgery. In addition to reflecting a greater confidence in biological drugs of paediatric gastroenterologists, this reveals a reluctance to refer young patients to surgery earlier in the course of their disease. Nonetheless, almost 50% of UC children who were managed medically were naïveto biological drugs (Table 3).

IPAA is the procedure of choice for the radical treatment of UC; it reduces the stool frequency when compared with straight ileoanal anastomosis [13] and dramatically diminishes the risk of cancer when compared with ileorectal anastomosis [14]. IPAA controls intestine-related EIMs and avoids prolonged drug administration. Moreover, since UC in children is—by definition—fated to be long-standing, eliminating the risk of neoplastic degeneration is crucial. Strict, invasive, and sometimes painful follow-ups are the price to pay for the retention of a diseased colon. However, the morbidity associated with the surgery is high, and complications occurring in the perioperative period may affect function in the long term [15, 16]. We included only patients operated on by a team led by a senior surgeon with a large pouch-procedure caseload and extensive experience with pelvic pouch surgery. When these conditions are met, no significant differences are to be expected (Table 1).

The incidence of pouchitis in adult UC patients reportedly ranges between 15% and 18% during the first year after ileostomy closure, reaching 48% by the tenth postoperative year [17, 18]. The first episode is observed within 1 year after ileostomy closure in 70% of patients with pouchitis [19]. The incidence may be even higher in children [20], with more than half developing recurrent episodes, and chronic pouchitis developing in up to 10% [21]. Failure occurs in almost 10% of these patients within 10 years [18, 22]. By using very stringent criteria, we were able to identify pouchitis in 25% and 12.5% of the children and adults, respectively; these proportions are lower than those reported in the literature. Pouchitis was managed by probiotics and antibiotic administration. The recurrence rates, rather than the incidence itself, appeared to differ between the groups, with all children experiencing a recurrence within 6 months. This discrepancy may be attributable to differences between children and adults in disease severity at presentation, as well as in their preoperative medical management.

Bowel function after IPAA is good in the large majority of patients and is stable with time over 20 years [23]. In the present series, despite the comparable overall number of evacuations per day between the adults and children, the latter had a lower mean number of evacuations per day per patient over a week. No differences were observed between patients according to age; however, there was a trend toward better discrimination and daytime continence among the younger patients.

Concerning health-related QoL, bowel control may be more important than stool frequency in children affected with UC. Uchida et al. [24] recently reported that children who experience good bowel function after IPAA may perceive their well-being to be identical to that of healthy individuals. A weak point of the QoL assessment in children with a pelvic pouch is the lack of standardized tools with which data is acquired. The wide time spans and data heterogeneity in most studies makes it difficult to interpret the data obtained in this population. In our practice, we routinely administer a QoL questionnaire to our patients. However, a statistical comparison between the QoL in adults and children was not performed in this series, since a child's perception of the world, life, death, and disease is quite different from that of adults.

IPAA resolves the interference between social functioning and the disruption of emotional status that is caused by refractory UC [25, 26]. We compared the bowel function of children who underwent IPAA and children whose UC was managed medically and found similar PedsQL-GSS scores in the two groups. This ruled out the risk of including control patients with unacceptably poor function (selection bias). We then evaluated QoL using the PedsQL SF-15. The results highlighted the superiority of IPAA over medical treatment in children suffering from UC. The difference was even more apparent on comparison of the QoL scores of the parents with those of their children, suggesting that parents of medically treated children are dealing with a feeling of uncertainty and fear due to potential relapse of the disease.

### 5.1. Study Limitations

The conclusions of this study should be considered in the light of several limitations. The follow-up period was short, although it was sufficient for our purposes and long-term results (i.e., pouch retention/failure) were not an aim of this research. The study may have lacked statistical power due to the smallness of the sample, which may have been responsible for the absence of significant differences. However, by considering bowel function after IPAA in paediatric versus adult UC patients as a primary aim, assuming success rates (i.e., good or optimal bowel function) of 90% and 80%, respectively, with a 20% noninferiority limit, 12 patients per group were required to be 70% sure that the upper limit of a two-sided 95% confidence interval would have excluded differences between groups (*α* = 0.05). Furthermore, the data were collected prospectively, and there is a dearth of evidence on this topic in the literature.

## 6. Conclusions

The findings of this study suggest that the functional results of IPAA are similar irrespective of whether this procedure is performed in childhood or adulthood. The health-related QoL is likely to be better after IPAA than for prolonged medical management. Such a beneficial effect was similarly observed for the parents' perception of their children's disease.

## Figures and Tables

**Table 1:** Characteristics of IPAA patients by age. Except where indicated otherwise, data are median (range) or *n* (%) values.

	IPAA childhood (*n* = 12)	IPAA adulthood (*n* = 24)	*P*
Age, years	12 (5–16)	33.5 (29–48)	**<0.0001**
BMI, kg/m^2^	18 (15–23)	21.5 (17–28)	0.09
Sex (male/female), *n *	5/7	11/13	>0.99
Steroids at time of first surgery*	4 (33.3)	9 (37.5)	>0.99
Azathioprine at time of first surgery	0 (0)	2 (8.3)	0.54
Naïve to biologic drugs	2 (16.6)	13 (54.2)	**0.04**
Drugs other than anti-UC drugs	1 (8.3)	7 (29.2)	0.2
EIMs	7 (58.3)	4 (16.7)	**0.02**
Three-stage procedure	5 (41.7)	4 (16.7)	0.12
Hand-sewn anastomosis	4 (33.3)	9 (37.5)	>0.99
Major perioperative complications	1 (8.3)	1 (4.2)	>0.99
Pouchitis			
At least one episode	3 (25)	3 (12.5)	0.38
Relapsing at least once	3/3 (100)	1/3 (33.3)	0.09

IPAA: ileal-pouch anal anastomosis; BMI: body mass index; UC: ulcerative colitis; EIMs: extraintestinal manifestations.

*>20 mg of corticosteroids.

**Table 2:** Bowel function of IPAA patients by age. Except where indicated otherwise, data are *n* (%) values.

Function	IPAA in childhood (*n* = 12)	IPAA in adulthood (*n* = 24)	*P*
Stool frequency per day, median (range)	5 (1–9)	5 (2–9)	0.54
Night evacuation	3 (25)	5 (20.8)	>0.99
Urgency	1 (8.3)	1 (4.2)	>0.99
Frequent incontinence during day	0 (0)	1 (4.2)	>0.99
Frequent incontinence during night	1 (8.3)	1 (4.2)	>0.99
Impaired discrimination	1 (8.3)	5 (20.8)	0.64

IPAA: ileal-pouch anal anastomosis.

**Table 3:** Characteristics of patients in the childhood by treatment. Except where indicated otherwise, data are median (range) or *n* (%) values.

	IPAA (*n* = 12)	Medical (*n* = 12)	*P*
Age, years	12 (5–16)	11 (6–16)	0.67
BMI, kg/m^2^	18 (15–23)	18 (15–21)	0.12
Sex (male/female), *n *	5/7	6/6	>0.99
Steroids at least once	5 (41.7)	4 (33.3)	>0.99
Azathioprine	1 (8.3)	2 (16.7)	>0.99
Naïve to biologic drugs	2 (16.6)	5 (41.7)	0.37
Drugs other than anti-UC drugs	1 (8.3)	2 (16.7)	>0.99
EIMs	7 (58.3)	2 (16.7)	0.08

IPAA: ileal-pouch anal anastomosis; BMI: body mass index; UC: ulcerative colitis; EIMs: extraintestinal manifestations.

## Abbreviations

EIMs: Extra-intestinal manifestations
GSS: Gastrointestinal Symptom Scale
IBDQ: Inflammatory bowel disease questionnaire
IPAA: Restorative proctocolectomy with ileal-pouch anal anastomosis
PedsQL: Pediatric Quality of Life Inventory
QoL: Quality of life
SF-15: Short Form-15
UC: Ulcerative colitis.
